# Circadian Clock and Nutrition

**DOI:** 10.3390/nu15092183

**Published:** 2023-05-04

**Authors:** Marian H. Lewandowski

**Affiliations:** Department of Neurophysiology and Chronobiology, Institute of Zoology and Biomedical Research, Jagiellonian University in Krakow, Gronostajowa 9, 30-387 Krakow, Poland; marian.lewandowski@uj.edu.pl

Rhythmicity is a fundamental characteristic of every living organism. It manifests itself at every level of an organisation, from structural and physiological changes to behaviour or cognitive processes. An evolutionarily dependent endogenous mechanism, in the form of a so-called biological clock, generates circadian changes in rhythmic processes independently of environmental conditions [1]. A very important property of this mechanism is its susceptibility to external synchronising influences. These not only allow the rhythms to biologically adapt to changing environmental conditions, but also to anticipate them, which is extremely important. The disruption of this biological balance between the internal circadian clock and rhythmically changing external conditions can and does cause disruptions (pathologia) in the functioning of the organism. One of the main synchronisers of the circadian clock is the changing day and night (light–dark) cycle, which is a consequence of the Earth’s rotational movement around its own axis, determining our most important rhythm of sleep and wakefulness [2]. The second very important so-called non-photic synchroniser of the biological clock, perhaps more important than light, is food. Its restrictive feeding (in the case of laboratory animals), or its regular intake by us, strongly influences the stability of the body’s rhythmic processes. For many years, a number of studies have shown that food, always taken at the same time of day, develops in our behaviour the anticipation of its administration time, otherwise known as intake, i.e., food anticipatory activity (FAA) [3]. The modern industrial world (modern society) forces us to be active 24/7, and this causes an unfavourable (‘incomprehensible’) change in the time of food intake for the body, which desynchronises biological rhythms, causing overweightness and obesity, and, as a consequence, many other diseases among civilisation, including the most dangerous ones. This is because the biological clock, by its very nature, needs more time to adapt (synchronise) to new conditions, including food, as opposed to the time in which they change.

This Special Issue in *Nutrients*, titled ‘Circadian Clock and Nutrition’, aims to present further scientific evidence on the interdependence of the biological clock and nutrition. The first experimental study conducted by Verde et al. [4] contains very interesting results on food preferences depending on our chronotype. Among other things, it confirmed previous observations that eating habits also depend on our chronotype, i.e., the endogenous properties of our biological clock [5]. The authors showed that overweight and obese subjects were mainly characterised by an evening chronotype. In addition to late (unhealthy) food intake, they also consumed food much more quickly (unhealthy) than people with the morning chronotype. In a second paper by the same research group [6], the authors examined the relationship between chronotypes and glycemic control, antidiabetic treatment, and the risk of complications in patients with type 2 diabetes (T2DM). According to the authors, the unhealthy eating habits of evening chronotype subjects may increase the risk of cardiometabolic complications, such as hypertension and ischaemic heart disease. Circadian food intake is regulated by a number of brain structures. One of these, referred to as the food-entrainable oscillator (FEO), which is very strongly associated with obesity, is the dorsomedial hypothalamus (DMH) [7]. In the study by Sanetra et al. [8], this structure was thoroughly examined for the first time using electrophysiology, immunofluorescence, as well as food intake and body weight gain assessment. A high-fat diet (HFD) given to rats for 4 weeks altered the circadian profile of the neuronal activity of this structure, making it high in the non-active phase of the animals. Thus, there was a shift in the phase of the animals’ feeding activity, by as much as 180°. Introducing a restriction of access to a high-fat diet (HFD), only to the time of day when the animals are active, restored normal DMH neuronal activity and feeding activity levels. The conclusions of this study confirm the importance of an appropriate time of day for food intake, which, even with a high-fat diet, does not necessarily cause side effects. The frequent and harmful disruption of the biological clock is the subject of Ren et al. [9]. The authors studied the effect of time-restricted feeding (TRF) on the effect of jet lag in mice. The authors found beneficial effects of TRF in some of the phase shifts that they used. Although behavioural fitness did not correlate well with the health of the studied mice, none of the TRF strategies they used worsened lipopolysaccharide-induced sepsis. Chrononutrition is a term in chronobiology that highlights the importance of eating at the right (appropriate) time. This is also very important in the proper functioning of the musculoskeletal system, the main system affecting our daily activities, including the all-important motor activity, the restriction of which affects our quality of life [10]. These very important physiological issues are addressed in the first of two Special Issue reviews [11]. The authors describe, among other things, nutritional strategies that may be potentially effective in maintaining homeostasis in the body. Indeed, movement, exercise, and regular physical activity are additional very important non-photic synchronisers of our biological clock. This is because the skeletal muscle diurnal clock plays an important role in lipid and glucose metabolism [12]. The authors encourage greater cooperation between dietitians and physiologists, especially those who deal with chrononutrition. This will allow for a better explanation and understanding of the physiological mechanisms of mutual interactions. It will also lead to the more effective prevention of diseases resulting from musculoskeletal disorders. The development of appropriate nutritional strategies safeguards the body’s energy homeostasis by providing, among other things, calcium and vitamin D, which are essential for healthy bones, preventing osteoporosis, the pathogenesis of which is also linked to changes in the circadian rhythm [13]. The review conducted by Erren et al. [14] was inspired by a Christmas card, painted by Colin Pittendrigh, sent to Jürgen Aschoff. Both gentlemen are pioneers of world chronobiology, and, although they were friends, their scientific discussions were very professional, interesting, and inspiring. I was lucky enough to witness a few. Based on the literature review, the authors analyse scientific research on nutrition, health, and disease during the Christmas and New Year period. What are the beneficial effects of chrononutrition over and after Christmas? An analytical study on carefully selected criteria that are used to evaluate our eating behaviour at Christmas time has definitely shown that holidays can have a detrimental effect on nutrition. The authors also point out that Christmas and New Year represent a time of many different resolutions, including dietary changes. This should be both absolutely and declaratively introduced, taking into account the results of chronobiological research on nutrition.

Therefore, to paraphrase the record in the Old Testament that “For everything there is a season and a time for every matter under heaven: a time to be born and a time to die; a time to plant and a time to pluck up what is planted. God has made everthing beautiful in its time”, it should also be assertively added that “there is also a time for eating”. This is the most general but very important conclusion of all the Special Issue articles in *Nutrients*.
